# Lats2 deficiency protects the heart against myocardial infarction by reducing inflammation and inhibiting mitochondrial fission and STING/p65 signaling

**DOI:** 10.7150/ijbs.84426

**Published:** 2023-07-03

**Authors:** Libao Liu, Shuai Huang, Yingzhen Du, Hao Zhou, Kai Zhang, Jinyuan He

**Affiliations:** 1 Department of Cardiothoracic Surgery, The Third Affiliated Hospital of Sun Yat sen University, Guangzhou, Guangdong, 510620, China.; 2 The Second Medical Center & National Clinical Research Center for Geriatric Diseases, Chinese PLA General Hospital, Medical School of Chinese PLA, Beijing, 100853, China.; 3 School of Medicine, University of Rochester Medical Center Rochester, Rochester, NY 14642, United States.

**Keywords:** Acute myocardial infarction, Lats2, mitochondrial fission, mtDNA, STING

## Abstract

Large tumor suppressor kinase 2 (*Lats2*) is a member of the Hippo pathway, a critical regulator of organ size. Since Lats2 activity may trigger mitochondrial dysfunction, a key pathogenic factor in acute myocardial infarction (AMI), this study sought to investigate whether *Lats2* deletion confers cardioprotection in AMI. AMI was induced in cardiomyocyte-specific *Lats2* knockout (*Lats2^Cko^*) and control (*Lats2^flox^*) mice. Twenty-eight days after AMI surgery, myocardial performance and mitochondrial homeostasis were impaired in *Lats2^flox^* mice. In contrast, *Lats2^Cko^* mice exhibited markedly preserved cardiac structure and contraction/relaxation activity, decreased fibrosis, reduced circulating cardiac injury biomarker levels, and enhanced cardiomyocyte viability. Consistent with these findings, siRNA-mediated *Lats2* silencing sustained mitochondrial respiration and inhibited apoptosis in hypoxia-treated HL-1 cardiomyocytes. Notably, *Lats2* deficiency inhibited AMI/hypoxia-related mitochondrial fission and inactivated STING/p65 signaling by preventing hypoxia-induced release of mtDNA into the cytosol. Accordingly, pharmacological reactivation of STING signaling abolished the cardioprotective effects of Lats2 ablation. Those data suggest that AMI-induced Lats2 upregulation is associated with impaired cardiomyocyte viability and function resulting from enhanced mitochondrial fission, mtDNA release, and STING/p65 pathway activation.

## Introduction

AMI is characterized by sudden blockade of blood supply to heart muscle after coronary thrombosis or spasm, resulting in massive cardiomyocyte death due to acute hypoxia 1. The pathogenesis of AMI is most usually linked to atherosclerosis, which involves plaque formation in the heart arteries that deliver blood to the cardiac myocyte. However, despite considerable research progress, the molecular mechanisms underlying AMI-mediated cardiomyocyte dysfunction and heart failure remain incompletely understood 2, 3. Primary therapies for AMI such as percutaneous coronary intervention (PCI), aimed at promptly opening the blocked coronaries to allow myocardial reperfusion. However, since ischemia-reperfusion injury greatly limits the efficacy of these cardioprotective treatments, exploring the molecular basis of AMI and designing new targeted therapies may provide additional clinical benefits.

Large tumor suppressor kinase 2 (Lats2) is a member of the Hippo pathway 4. Recent findings have highlighted the indispensable action of Lats2 on regulating cellular proliferation, differentiation, and apoptosis in both normal and cancer cells 5, 6. Lats2 transcription and expression levels are highly sensitive to tumor suppressive signaling 5, modulate mitochondria-related apoptotic pathways 7, and influence cancer metastasis and invasion 8. Recent studies have also associated Lats2 with the development of many cardiovascular disorders. During a pressure overload-caused heart failure, elevated Lats2 expression promotes the abundance of pro-death factors, such as Bax and Bak, leading to cardiomyocyte death mediated by the p53 pathway 9. Similarly, Lats2-mediated mitochondrial injury and cardiomyocyte dysfunction have been identified as key downstream effectors of abnormal inflammatory responses in septic cardiomyopathy 10. However, evidence describing the influence of Lats2 in AMI is limited.

Previous studies have reported three primary pathological effects of Lats2 activation on mitochondrial biology: i) induction of mitochondrial fission 10; ii) activation of the mitochondrial pathway of apoptosis 11; and iii) disruption of mitochondrial metabolism 12. Since these three molecular events have been proposed as potential factors instigating AMI progression, it is reasonable to hypothesize that Lats2-mediated mitochondrial dysfunction may also play a role in AMI. Excessive mitochondrial fission has been found to promote mitochondrial membrane rupture, with subsequent release of mitochondrial DNA (mtDNA) outside of mitochondria 13-15. The latter was shown to target the stimulator of interferon genes (STING)/p65 pathway, leading to an inflammatory response 16-18. Despite the above evidence, the pathological impact of mitochondrial fission as a trigger of mtDNA-mediated STING/p65 signaling in AMI remains unexplored. Therefore, in this work we employed an AMI mouse model, as well as hypoxia-treated cultured cardiomyocytes, to determine whether Lats2 contributes to AMI-related myocardial dysfunction by inducing mitochondrial fission, mtDNA release, and STING/p65 pathway activation.

## Materials and Methods

### Animals

Lats2-floxed (Lats2*^flox^*) mice (strain #027934) and α-MHC (alpha myosin heavy chain)-Cre transgenic (α-MHC*^Cre^*) mice (strain #011038) were obtained from The Jackson Laboratory and crossed to obtain cardiomyocyte-specific Lats2-knockout (Lats2*^Cko^*) mice. Lats2*^flox^* mice served as control. Lats2 deficiency efficiency was confirmed by qPCR. Sample sizes were estimated based on sample size power calculations using pilot experiment data.

### Myocardial infarction mouse model and treatments

AMI was induced by permanent ligation of the left anterior descending (LAD) coronary artery 19. Sham operations were performed without the ligation of LAD artery. The procedure was performed by an investigator who used the ear-tag number as identification and was blinded to the treatment of animals. STING agonist-1 (G10, Cat. No. S8954, Selleck) (1 μg per mouse) or PBS (control) was injected intraperitoneally 15 min prior to AMI modeling and sustained for 7 days. A minimum of 6 animals per group were used. Four mice died during surgery or post-operative recovery prior to end point determinations and were thus excluded. All 18 surviving mice were used in this study.

### Echocardiography

Echocardiography was conducted 28 days after AMI/sham surgery using a Vevo 2100 instrument (Visual Sonics, Toronto) by an operator blinded to the treatment group 20. The parasternal long-axis view was recorded. By tracing the endocardial borders at end-diastole and end-systole, quantitative information on systolic and diastolic performance 21.

### Histological analyses and electron microscopy

Infarct size and tissue fibrosis were measured 28 days after AMI induction. Hearts were excised and 5-mm sections were cut and stained with HE 22. To quantify myocardial fibrosis, heart samples were fixed with 10% formalin, and submitted for Masson's trichrome staining. Collagen volume was determined by quantitative morphometry of mid-LV sections 23. Electron microscopy was conducted as previously described 24.

### qPCR

RNA was collected 25 after disrupting the samples with a TissueLyser LT bead mill (Qiagen). Subsequently, the mRNA in 1 µg of RNA was transcribed into cDNA 26. The primers used in the present study were shown in Supplemental Information.

### Cell culture and treatments

HL-1 cells were purchased from Sigma-Aldrich (Cat. No. SCC065) and cultured under normal conditions. To mimic myocardial infarction *in vitro*, cells were treated to hypoxia condition (94% N_2_, 1% O_2_, 5% CO_2_) for 48 h 27. Cells were preincubated for 1 h with Mdivi-1 (5 mM, Cat. No. S7162, Selleck Chemicals, Houston, TX, USA) before hypoxia. To activate the STING pathway, HL-1 cells were similarly pre-treated with a STING agonist (G01, 10 μM, Cat. No. S8954, Selleck Chemicals) for 2 hrs. FCCP (5mM) was applied to activate mitochondrial fission 28.

### ROS, mitochondrial potential, and oxygen consumption rate (OCR) detection

Cellular ROS generation was evaluated using 6-carboxy-2',7'dichlorodihydrofluorescein diacetate (DCFDA) 29. HL-1 cells were treated with 20 µM DCFDA at 37°C for 30 min. After two washes in Tyrode's buffer, fluorescence was measured at 488 nm excitation/530 nm emission by confocal microscopy (Nikon, Japan). Mitochondrial potential was detected with the assistance of the JC-1 probe (Cat. No. T3168, ThermoFisher). Mitochondrial OCR was measured as previously described 30.

### Detection of cardiac injury markers, apoptosis-related markers, ATP, and glucose

Sera from mice were separated from peripheral blood by centrifugation and and lactate dehydrogenase (LDH), creatine kinase MB (CK-MB), and troponin T (TnT), concentrations were determined by ELISA kits (Mouse TnT, Cat. No. abx585262, Abbexa, Cambridge, UK; Mouse CK-MB, Cat. No. ab285231, Abcam, Cambridge, UK; Mouse LDH, Cat. No. abx154299, Abbexa). For determination of apoptosis-related markers and variables related to mitochondrial function in cultured HL-1 cells, ELISA kits were used to measure caspase-9 and ATP levels (Mouse Caspase 9 ELISA Kit, Cat. No. abx255241, Abbexa), whereas fluorometric/colorimetric assays were used to measure caspase-3 activity (Caspase-3 Activity Assay, Cat. No. 5723, CST, Danvers, MA, USA) and glucose levels (Glucose Assay Kit, Cat. No. ab65333, Abcam) 31. Th levels of ATP in HL-1 cells were measured by an ELISA kit (Mouse Adenosine Triphosphate ELISA Kit, Cat. No. MBS724442, MyBioSource.) 32.

### Cell viability assay

Cell viability was assessed using Cell Counting Kit 8 (Cat. No. ab228554, Abcam) and an LDH Assay Kit (Cytotoxicity) (Cat. No. ab65393, Abcam) in accordance with the supplier's protocols 33.

### Immunoblot analysis

Frozen hearts and/or cells were lysed in 1 ml ice-cold RIPA buffer with protease inhibitor cocktail mixture, mixed with 1 mm and 3 mm ceramic beads, and homogenized with a Bertin Precellys 24 device at 5,000 rpm for 10 s with six repeats. The lysates were centrifuged at 5,000 g for 15 min at 4 °C 34. After removing the upper aqueous phase and bottom organic phase containing lipids, the protein pellets were washed once with ice-cold methanol and solubilized by sonication in SDS-lysis buffer containing protease inhibitor mixture 35. For detecting membrane proteins, sample was mixed with 4x loading buffer and incubated at 37 °C for 20 min before SDS-PAGE. Primary antibodies included: cGAS (Cat No. ab252416, Abcam), STING (Cat No. ab288157, Abcam), phos-p65 (Cat No. ab76302, Abcam), and p65 (Cat No. ab32536, Abcam).

### Immunofluorescence

The 4% paraformaldehyde, 0.1% Triton X-100/PBS, and 1% bovine serum albumin were used for immunofluorescence. Then, cells were treated with primary antibodies (cyt-c, Cat. No. ab76107, Abcam; phos-p65, Cat. No. ab131100, Abcam), followed by treatment with secondary antibodies (Donkey Anti-Rabbit IgG H&L Alexa Fluor® 647 preadsorbed, Cat. No. ab150063, Abcam) 36.

### siRNA transfection

Silencing of Lats2 gene was induced through the small interfering RNA (siRNA) technique. Silencer Select Lats2-specific siRNA and negative control siRNA were purchased from Thermo Fisher Scientific (Cat. No. AM16708) 37. siRNAs were used to incubate with HL-1 cells with the assistance of Lipofectamine RNAiMAX (Thermo Fisher Scientific).

### Statistical data

Statistical data were analyzed using GraphPad Prism version 8.0. Data are expressed as mean ± SEM. Experiments were performed with 2-3 technical replicates of each sample, and the resulting data was averaged for each biological replicate. ANOVA followed by Tukey's multiple comparison test were used to analyze the differences between two or more groups. P < 0.05 was considered significant.

## Results

### *Lats2* deficiency in heart reduces AMI-related myocardial dysfunction

**Figure 1:**
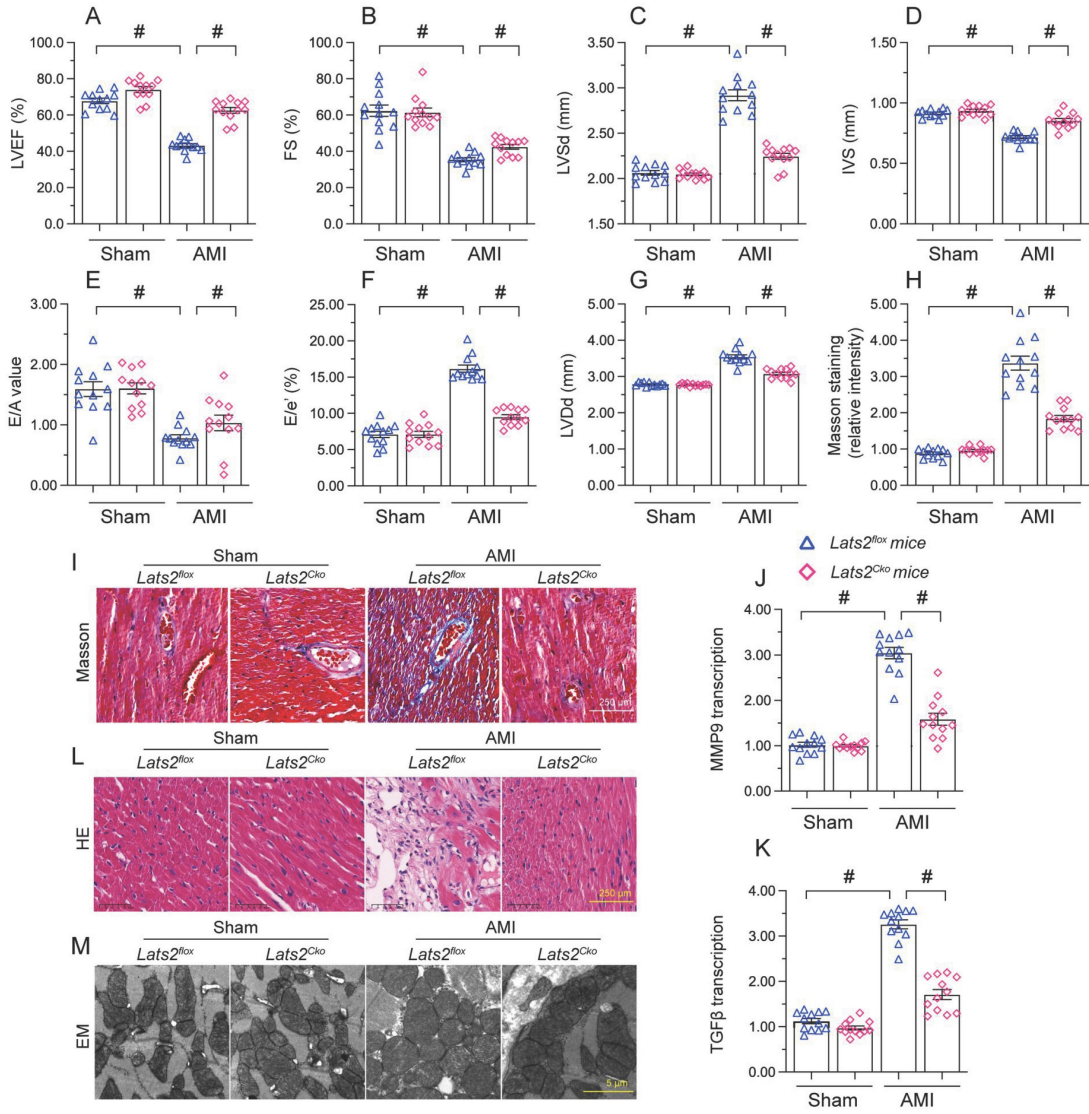
** Lats2 knockout alleviates myocardial dysfunction in an AMI mouse model.** *Lats2^Cko^* and *Lats2^flox^* mice were subjected the permanent ligation of the LAD coronary artery to induce AMI. **(A-G)** Myocardial function was determined 28 days following AMI surgery using echocardiography. Left ventricular ejection fraction (LVEF), fractional shortening (FS), left ventricular systolic dimension (LVSd), left ventricular diastolic dimension (LVDd), early to late (atrial) mitral flow velocity ratio (E/A), ratio of mitral peak velocity of early filling to early diastolic mitral annular velocity (E/e′), and interventricular septal thickness (IVS) were measured in *Lats2^Cko^* and *Lats2^flox^* mice. **(H, I)** Masson's staining was used to observe myocardial fibrosis. **(J, K)** RT-qPCR was used to analyze cardiac transcription of MMP9 and TGFβ. **(L)** HE staining was used to observe myocardial structure. **(M)** Electron microscopy (EM) was used to observe the ultrastructure of myocardium. #p<0.05.

To describe the pathological feature of Lats2 in AMI, we modeled AMI in cardiomyocyte-specific *Lats2* knockout (*Lats2^Cko^*) and control (*Lats2^flox^*) mice through permanent ligation of LAD. Echocardiographic evaluation of myocardial function 28 days after AMI induction (Figure 1A-1G) indicated a drop in the myocardial contraction parameters LVEF and LVEF in *Lats2^flox^* compared to sham-operated mice. Suggesting dilation of the heart cavity, IVS was decreased and LVDs/LVDd was increased after AMI in *Lats2^flox^* mice. In addition, disrupted cardiac relaxation capacity, evidenced by decreased E/A and E/e', was also observed in these mice. Interestingly, following AMI the contractile function of the heart was normalized in *Lats2^Cko^* mice (Figure 1A-1G). Masson's trichrome staining evaluation of AMI-related cardiac fibrosis showed that AMI Lats2*^flox^* mice developed severe fibrosis, and this alteration was significantly attenuated in *Lats2^Cko^* mice (Figure 1H, 1I). Supporting these findings, the transcription of pro-fibrosis genes, i.e. MMP9 and TGFβ, was significantly elevated in *Lats2^flox^* mice and maintained instead at near normal levels in *Lats2^Cko^* mice (Figure 1J, 1K). HE staining further showed that AMI-related myocardial swelling and dilation were largely reduced in *Lats2^Cko^* mice compared to *Lats2^flox^* mice (Figure 1L). Similarly, electron microscopy (EM) showed that after AMI, myocardial fiber disarray, evidenced by blurred Z-lines, was obvious in *Lats2^flox^* mice but markedly attenuated in *Lats2^Cko^* mice (Figure 1M). These results showed that cardiac-specific *Lats2* deletion markedly attenuates AMI-induced myocardial deficits in mice.

### Loss of Lats2 attenuates AMI-induced cardiomyocyte damage

**Figure 2:**
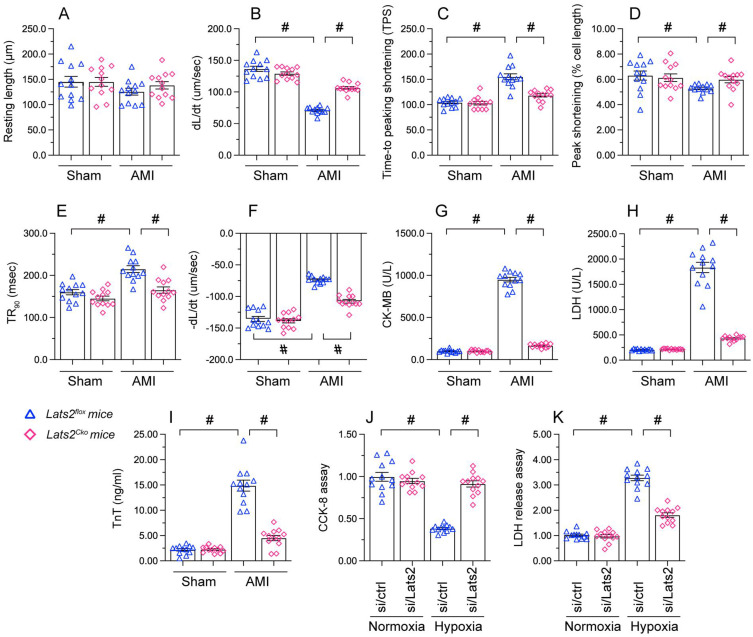
** Loss of Lats2 attenuates cardiomyocyte damage and dysfunction induced by myocardial infarction. (A-F)** Contractility measurements in primary cardiomyocytes isolated from *Lats2^Cko^* and *Lats2^flox^* mice. PS, peaking shortening; +dL/dt, maximal velocity of shortening; TPS, time-to-peak shortening; -dL/dt, maximal velocity of relengthening; TR90, time-to-90% relengthening. **(G-I)** Serum was collected from mice and levels of TnT, CK-MB, and LDH were measured via ELISA. **(J)** Cell viability was determined using CCK-8 assays in HL-1 cells transfected with siRNA against Lats2 (Lats2/siRNA) or negative control siRNA and exposed to hypoxia for 48 h. **(K)** LDH levels in the supernatant of hypoxia-treated HL-1 cells were determined by ELISA. #p<0.05.

To investigate the mechanisms by which Lats2 deficiency alleviates AMI-related cardiac dysfunction, we evaluated the features of cardiac myocytes obtained 28 days after AMI induction from *Lats2^flox^* and *Lats2^Cko^* mice. Results of morphometric analyses, summarized in Figure 2A-2F, indicated that *Lats2* deletion did not alter cardiomyocyte structure nor average length. In contrast, both peak shortening and maximal shortening velocity were reduced, whereas time-to-peak shortening was prolonged, in cardiac myocytes obtained from *Lats2^flox^* mice. Notably, these parameters were corrected in cardiac myocytes from* Lats2^Cko^* mice. Likewise, significant suppression of the maximal velocity of relengthening and time-to-90% relengthening were evident in *Lats2^flox^* cardiac myocytes, and these alterations were largely prevented in *Lats2-*deficient cardiomyocytes (Figure 2A-2F). In accordance with the cardioprotective effects of *Lats2* ablation *in vivo*, after AMI induction serum biomarkers of cardiomyocyte rupture, namely TnT, CK-MB, and LDH, were markedly elevated in *Lats2^flox^* mice but not in *Lats2^Cko^* mice (Figure 2G-2I).

To focus on the cardioprotective properties of *Lats2* deletion on cardiomyocyte dysfunction, cultured mouse HL-1 cardiomyocytes were treated with hypoxia. To induce Lats2 silencing, siRNA against Lats2 (Lats2/siRNA) was infected into HL-1 cells before exposure to hypoxic stress. After 48 h of hypoxia, cardiomyocyte function was detected by CCK-8 and LDH analysis experiments. Hypoxia treatment reduced viability (Figure 2J) and promoted LDH upregulation in HL-1 cells infected with negative control siRNA (Figure 2K). On the other hand, cell viability was preserved, and LDH release was prevented, in cells treated with Lats2/siRNA (Figure 2J, 2K). Those data offered obvious evidence that *Lats2* deficiency ameliorates AMI-mediated cardiomyocyte dysfunction.

### Lats2 silencing prevents mitochondrial dysfunction in hypoxic cardiomyocytes

**Figure 3:**
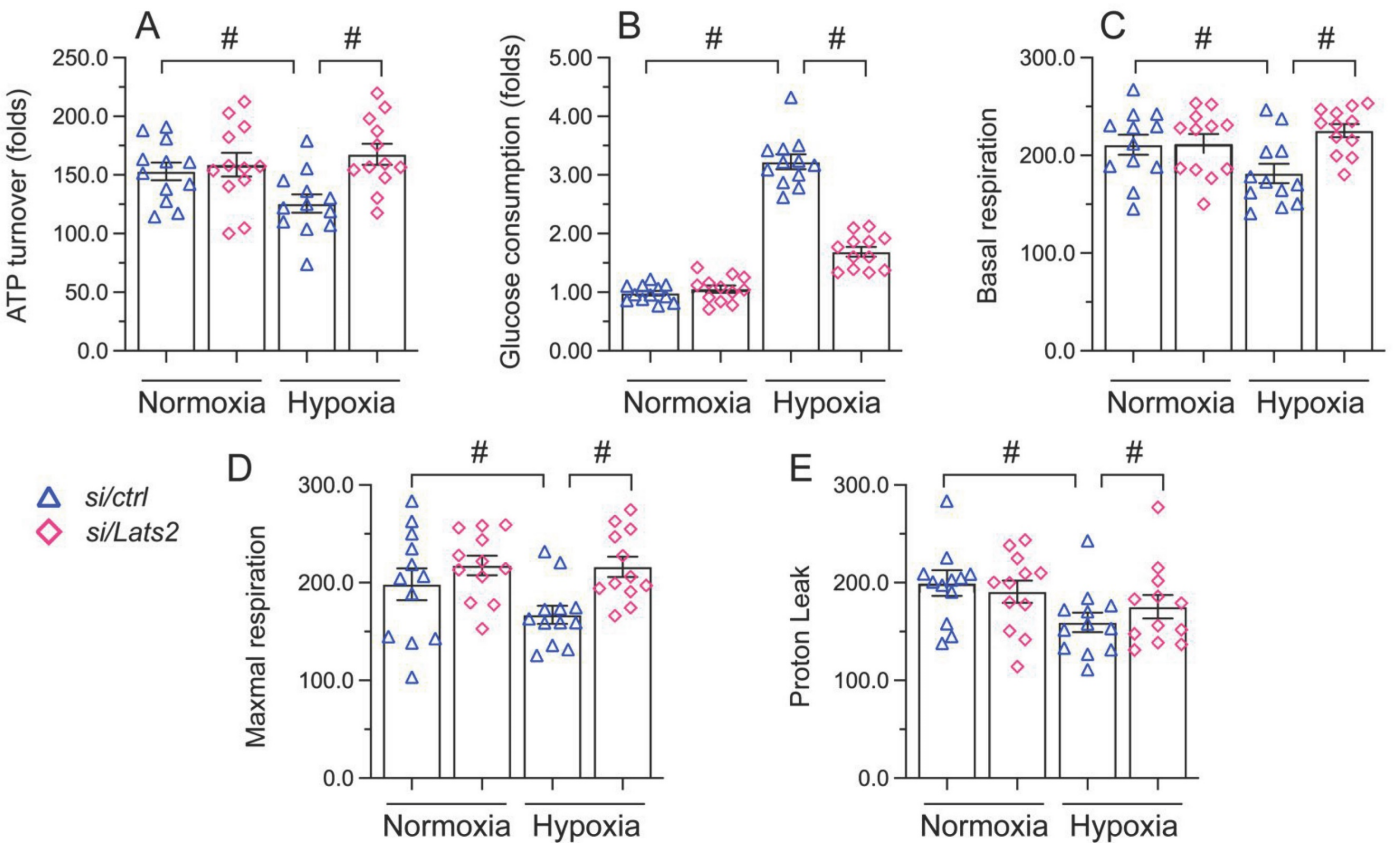
** Lats2 silencing prevents mitochondrial dysfunction in hypoxic cardiomyocytes *in vitro*. (A)** ATP production was measured by ELISA in HL-1 cells transfected with Lats2/siRNA or control siRNA before hypoxia exposure for 48 h. **(B)** Glucose levels in culture media from HL-1 cells were measured by ELISA. **(C-E)** OCR and gene expression analysis of mitochondrial respiration function in HL-1 cells. #p<0.05.

Mitochondrial damage and dysfunction are key determining events in AMI-related cardiac arrest 38, 39. Since Lats2 acts as a negative regulator of mitochondrial fitness 10, 11, we asked whether Lats2 deficiency would attenuate mitochondrial dysfunction in hypoxia-treated HL-1 cardiomyocytes. ELISA measurements showed that ATP production was repressed in cells administrated with negative control siRNA, but restored instead in those transfected with Lats2/siRNA (Figure 3A). Furthermore, accelerated glucose consumption was evident in the latter group of cells (Figure 3B). Parallel measurements of oxygen consumption rate (OCR) in isolated mitochondria indicated that following hypoxia, mitochondrial respiration was inhibited in control siRNA-delivered cells, but not in Lats2/siRNA-infected cells (Figure 3C-3E). The above data showed that Lats2 deletion sustains mitochondrial function and homeostasis in cardiac cells exposed to hypoxic stress.

### Lats2 silencing inhibits mitochondria-dependent apoptosis in hypoxic cardiomyocytes

**Figure 4:**
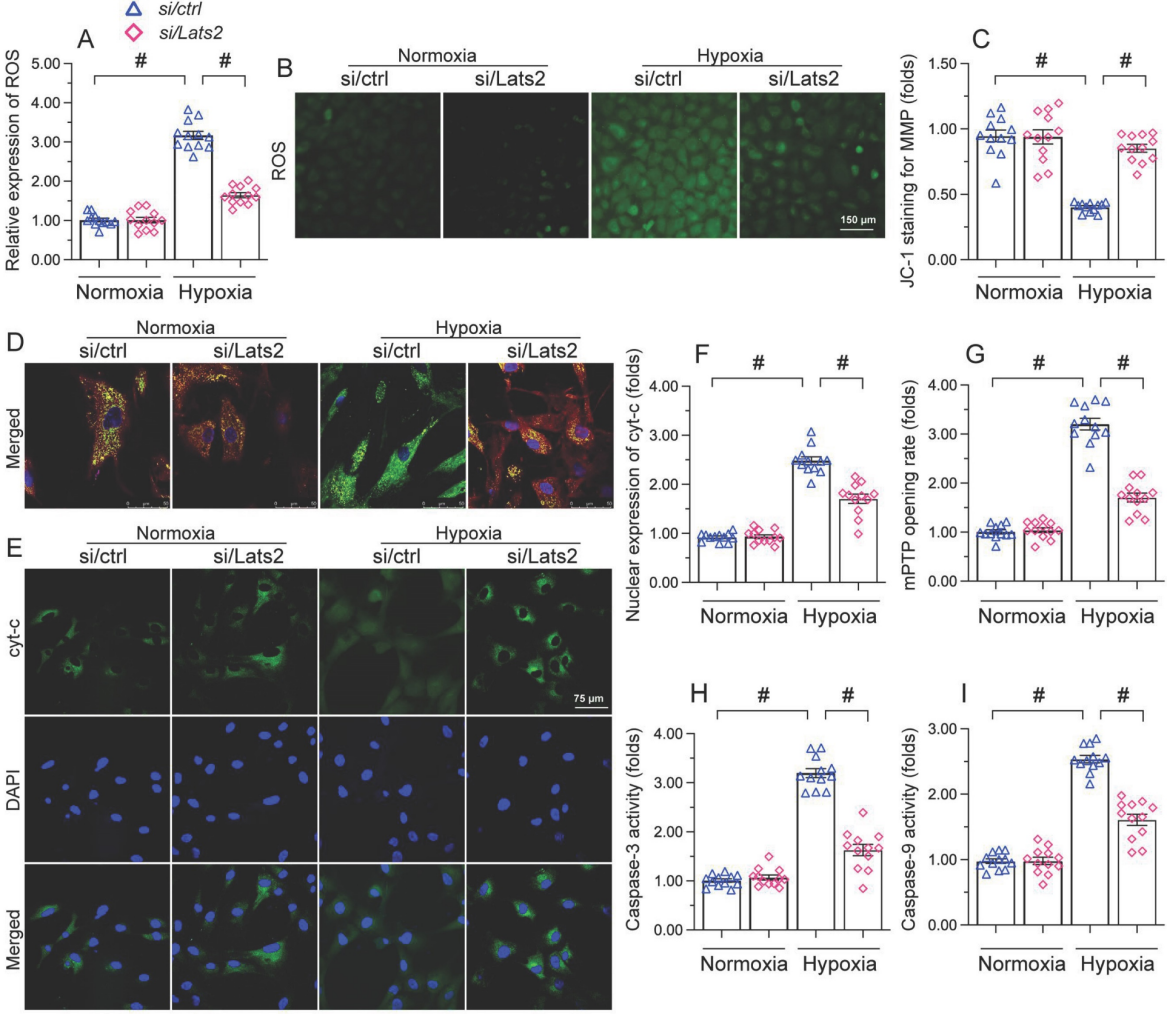
** Lats2 silencing prevents ROS generation, mitochondrial membrane depolarization, and mitochondria-dependent apoptosis in hypoxic cardiomyocytes.** Measurements were made in cultured HL-1 cardiomyocytes 48 h after hypoxia treatment.** (A, B)** DCFDA fluorescence assay was used to determine the production of cellular ROS. **(C, D)** Analysis of changes in mitochondrial membrane potential (ratio of red-to-green fluorescence intensity) in cells loaded with JC-1. **(E, F)** Immunofluorescence staining of cyt-c. **(G)** Measurement of mPTP opening rate. **(H, I)** Analysis of caspase-3 activity and caspase-9 concentration. #p<0.05.

Since AMI-related mitochondrial dysfunction correlates with cardiomyocyte apoptosis 40-42, we next asked whether Lats2 deficiency would also prevent mitochondria-dependent apoptosis in hypoxic cardiomyocytes. Oxidative stress is regarded as an early signal of activation of the mitochondria-initiated apoptotic cascade 43. As illustrated in Figure 4A, 4B, hypoxia augmented the reactive oxygen species (ROS) production in control HL-1 cardiomyocytes, and this effect was nullified by Lats2/siRNA. Research has shown that oxidative stress is paralleled by mitochondrial potential reduction 44, which augments the release of pro-death factors, such as cyt-c and Smac. JC-1 fluorescence assays showed that in response to hypoxic stress, mitochondrial membrane potential was reduced in control cells, but remained instead stable in those transfected with Lats2/siRNA (Figure 4C, 4D). In turn, immunofluorescence showed that hypoxia promoted cytosolic and nuclear redistribution of cyt-c in control-siRNA cells (Figure 4E, 4F). In contrast, transfection of Lats2/siRNA normalized the subcellular distribution of cyt-c and prevented its translocation to cell nuclei (Figure 4E, 4F). Cyt-c release is facilitated mitochondrial permeability pore (mPTP) opening and triggers the intrinsic apoptosis pathway through caspase-9/3 activation. Additional assays showed that in control cells the mPTP opening rate was prolonged upon hypoxia (Figure 4G), and this alteration was accompanied by increased caspase-9 levels and activation of caspase-3 (Figure 4H, 4I). In contrast, a normalized mPTP opening rate (Figure 4G), as well as reduced caspase-9/3 expression/activity (Figure 4H, 4I) were noted in cardiomyocytes administrated by Lats2/siRNA. Thus, Lats2 signaling contributes critically to mitochondria-dependent apoptosis in hypoxia-exposed cardiomyocytes.

### Lats2 ablation reduces mitochondrial fission in hypoxic cardiomyocytes

**Figure 5:**
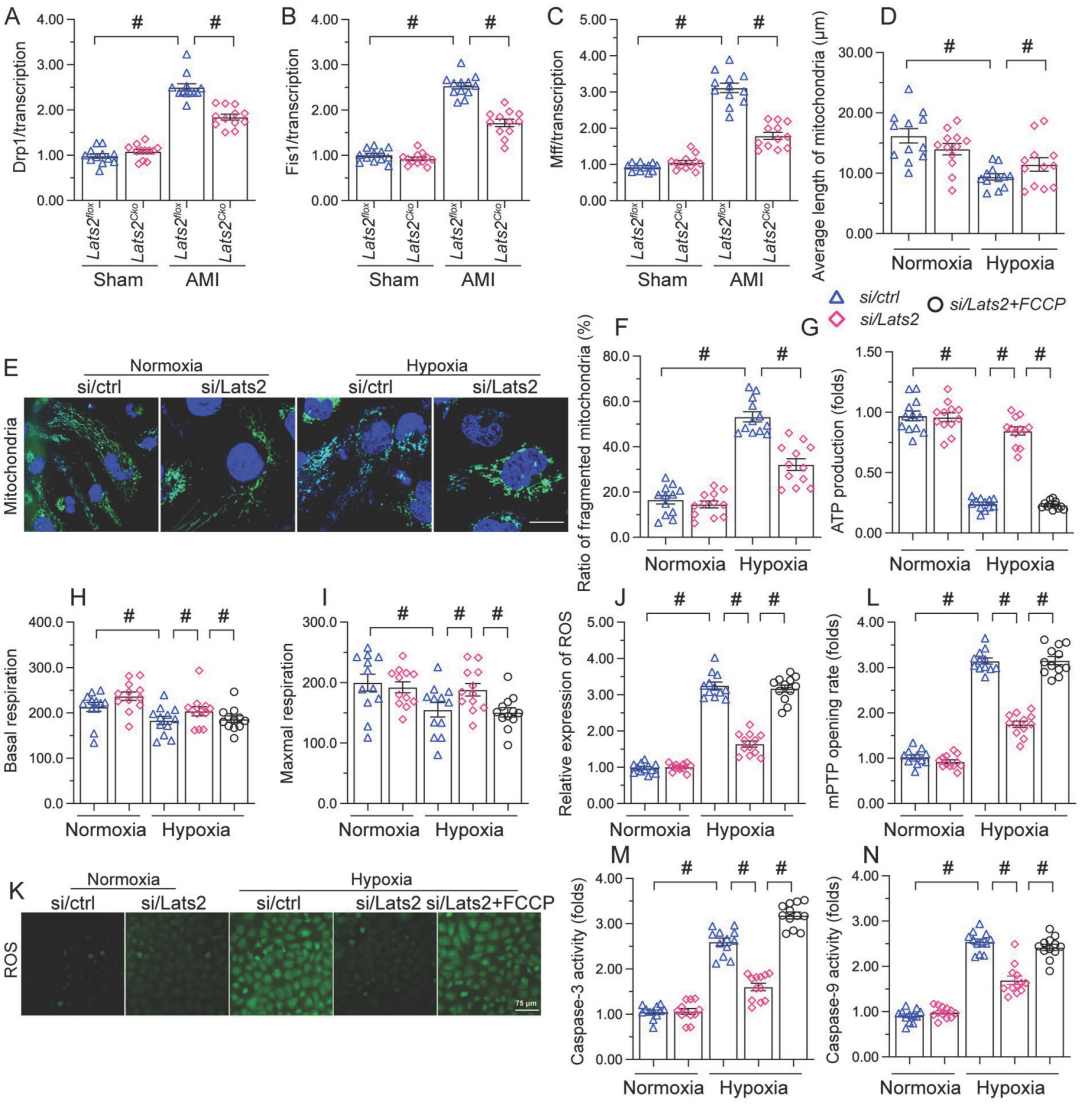
** Ablation of Lats2 attenuates myocardial infarction-mediated mitochondrial fission. (A-C)** RT-qPCR analysis of the transcription of *Drp1*, *Mff*, and *Fis1* in mouse heart. **(D-F)** Immunofluorescent detection of mitochondria and quantification of mitochondria average length and ratio of HL-1 cells with fragmented vs tubular mitochondria. **(G)** ELISA-based analysis of ATP production in HL-1 cells pre-treated with FCCP. **(H, I)** Analysis of mitochondrial respiration function in HL-1 cells pre-treated with FCCP. **(J, K)** Analysis of ROS generation in FCCP pre-treated, DCFDA-loaded HL-1 cells. **(L)** Quantification of mPTP opening time in HL-1 cells pre-treated with FCCP. **(M, N)** Analysis of caspase 3 activity and caspase 9 concentration in FCCP-treated HL-1 cells. #p<0.05.

Whereas previous studies have shown that Lats2 signaling promotes mitochondrial fission in cancer cells 10, this phenomenon has not been verified in cardiac tissue following AMI. RT-qPCR displayed that compared with sham-operated mice, the mRNA abundance of mitochondrial fission-related genes (*Drp-1*, *Mff*, and *Fis1*) was doubled after AMI in heart tissue from *Lats2^flox^* mice (Figure 5A-5C). Similarly suggesting mitochondrial fission induction, upon hypoxic exposure the mitochondrial network was disrupted and fragmented mitochondria were observed in cultured HL-1 cardiomyocytes (Figure 5D-5F). Concurrent morphometric analysis showed that hypoxia treatment shortened the mitochondrial length from ~17 μm to 9 μm (Figure 5D-5F). In contrast, loss of Lats2 prevented the upregulation of mitochondrial fission-related genes *in vivo* (Figure 5A-5C) and normalized mitochondrial network and morphology in HL-1 cells (Figure 5D-5F).

To confirm that Lats2-mediated mitochondrial fission contributes to hypoxia-related mitochondrial dysfunction, FCCP, an activator of mitochondrial fission, was incubated with HL-1 cardiomyocytes. After hypoxia induction, the normalizing effects of Lats2/siRNA transfection on ATP production (Figure 5G), mitochondrial respiration (Figure 5H, 5I), and oxidative stress (Figure 5J, 5K) were significantly attenuated or abolished by FCCP. Similarly, upon FCCP treatment, Lats2 silencing no longer prevented abnormal mPTP opening (Figure 5L) nor caspase-9/3 activation (Figure 5M, 5N) in hypoxia-treated cardiomyocytes. These data suggest that during AMI, Lats2-dependent mitochondrial fission contributes to impaired mitochondrial function in cardiomyocytes.

### AMI-induced, Lats2-dependent mitochondrial fission activates the mtDNA/STING/p65 pathway in cardiomyocytes

**Figure 6:**
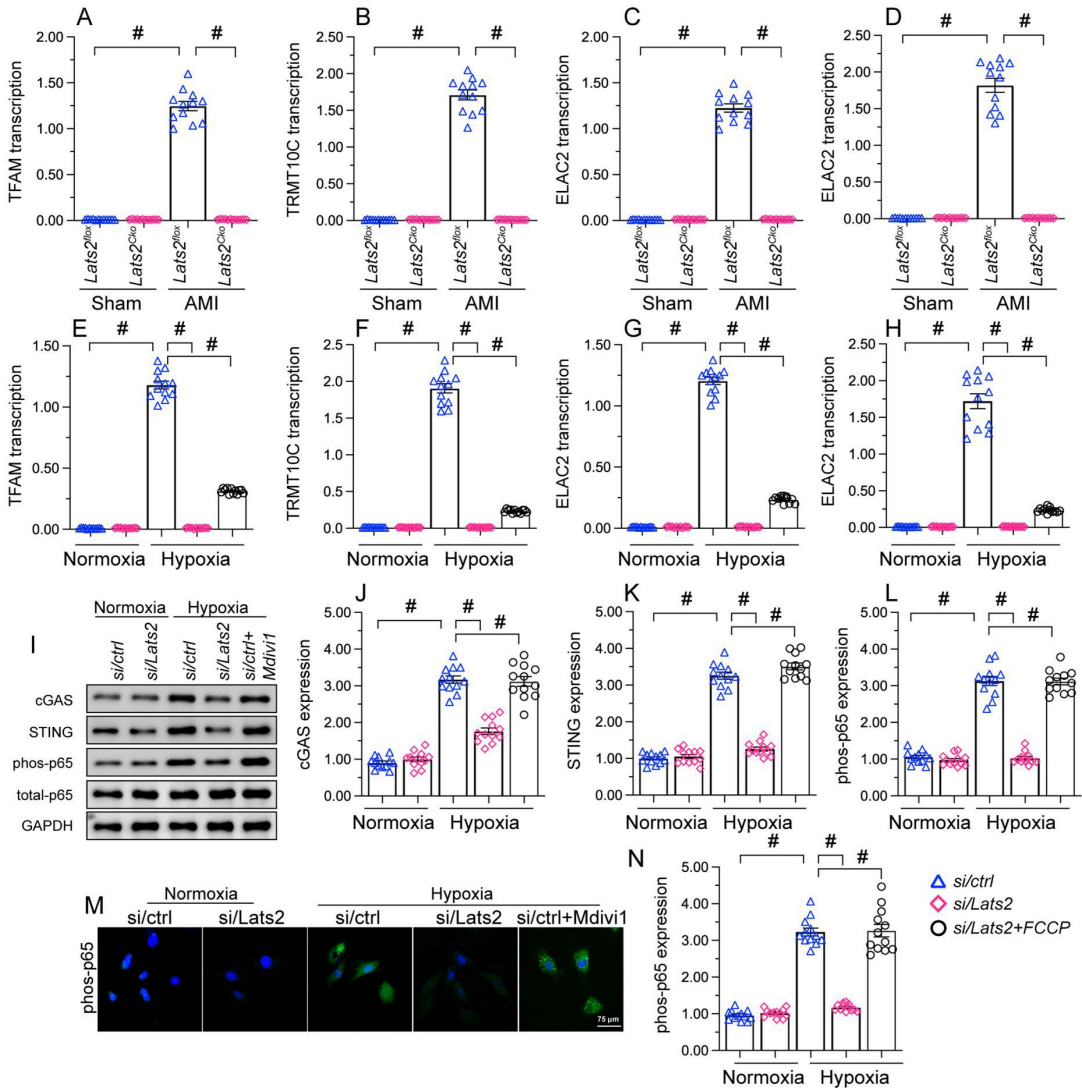
** Excessive mitochondrial fission activates the mtDNA/STING/p65 pathway in cardiomyocytes. (A-D)** RT-qPCR was used to analyze the transcription of *TFAM*, *TRMT10C*, *ELAC2*, and *FASTKD2* in cytosolic extracts from heart tissues. **(E-H)** Transcriptional analysis of *TFAM*, *TRMT10C*, *ELAC2*, and *FASTKD2* expression in cytosolic fractions from HL-1 cells.** (I-L)** Western blot analysis of cGAS and STING expression and p65 phosphorylation in HL-1 cells. **(M, N)** Immunofluorescence detection of phosphorylated p65 in HL-1 cells. Mdivi-1 was used to inhibit mitochondrial fission before hypoxia treatment. #p<0.05.

To further explore the outcomes of enhanced cardiac mitochondrial fission triggered by AMI, we focused on mtDNA leakage and the activation of STING/p65 pathway. To assess mtDNA leakage, we analyzed the expression of mtDNA-specific mRNAs in the mitochondria-free cytosolic fraction of cardiomyocytes. The expression of *TFAM*, *TRMT10C*, *ELAC2*, and *FASTKD2* mRNAs was undetectable in normal heart tissue (Figure 6A-6D). Upon AMI induction, the expression of these mtDNA-related mRNAs was prominent in heart samples from *Lats2^flox^* mice, and partly suppressed in *Lats2^Cko^* mice (Figure 6A-6D). Similar results were obtained *in vitro*, in hypoxic cardiomyocytes (Figure 6E-6H). Importantly, further experiments in HL-1 cells showed that supplementation of Mdivi-1 reduced the abundance of mtDNA-related mRNAs in the cytosolic compartment (Figure 6E-6H). These results suggested that under hypoxic conditions, Lats2-mediated mitochondrial fission facilitates mtDNA leakage into the cytosol.

Western blots further showed that following hypoxia, expression levels of cGAS, STING, and phosphorylated p65 were markedly elevated in siRNA-control cells, and these changes were prevented in Lats2-deficient cells (Figure 6I-6L). Consistent with these findings, immunofluorescence showed that hypoxia led to increased p65 phosphorylation, and this alteration was corrected by Lats2/siRNA or Mdivi-1 pre-treatment (Figure 6M, 6N). These results suggested that Lats2-mediated mitochondrial fission leads to cGAS/STING/p65 pathway activation in heart tissue during AMI.

### STING reactivation attenuates the cardioprotective action of Lats2 ablation in AMI

**Figure 7:**
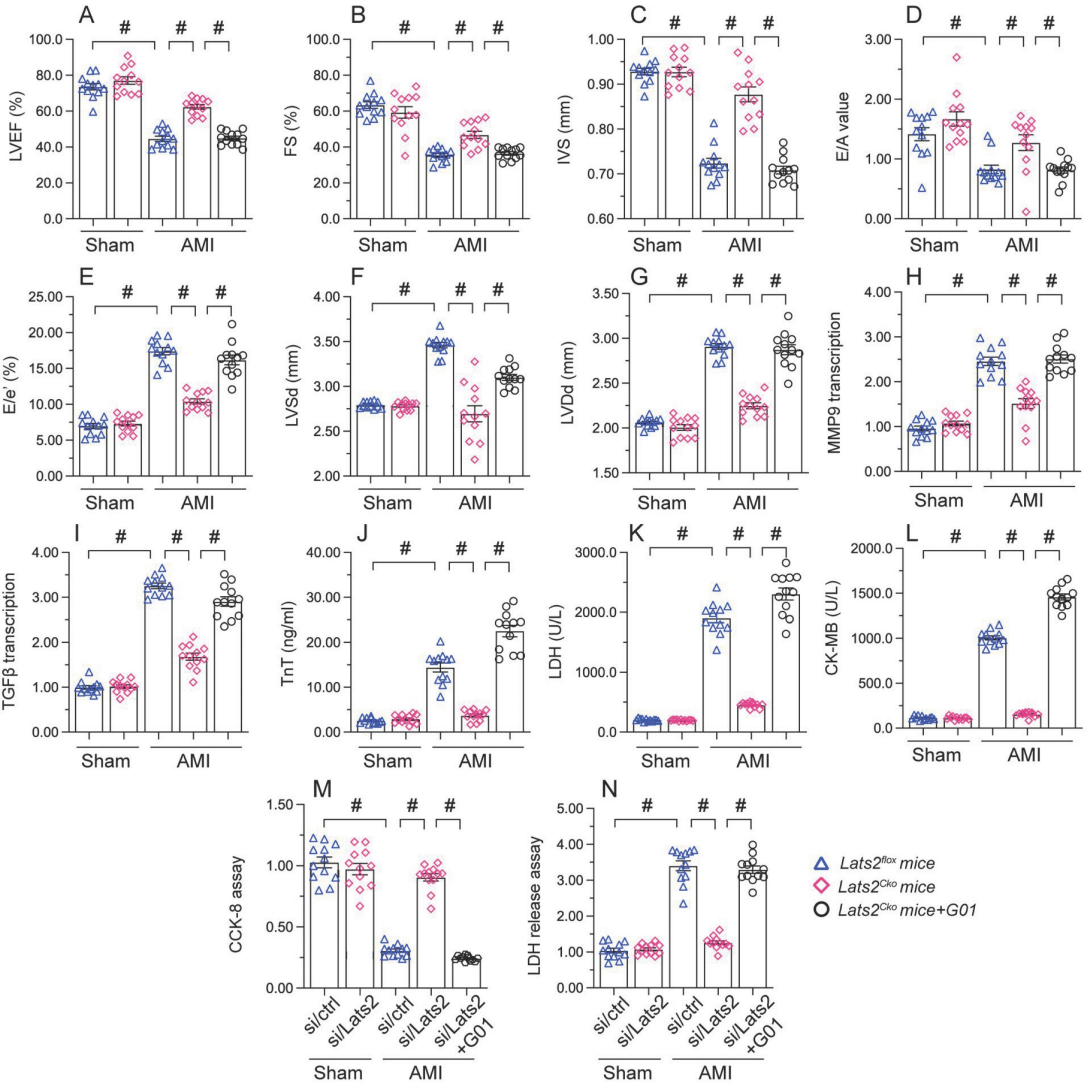
** Re-activation of STING abolishes the cardioprotective effects of Lats2 ablation in AMI. (A-G)** Myocardial function was determined by echocardiography 28 days after AMI surgery in *Lats2^Cko^* and *Lats2^flox^* mice treated or not with STING agonist-1 (G10). Left ventricular ejection fraction (LVEF), fractional shortening (FS), left ventricular systolic dimension (LVSd), left ventricular diastolic dimension (LVDd), early to late (atrial) mitral flow velocity ratio (E/A), ratio of mitral peak velocity of early filling to early diastolic mitral annular velocity (E/e′), and interventricular septal thickness (IVS) were measured in *Lats2^Cko^* and *Lats2^flox^* mice.** (H, I)** RT-qPCR was used to analyze the transcription of MMP9 and TGFβ in heart tissue from *Lats2^Cko^* and *Lats2^flox^* mice. **(J-L)** Serum was collected from mice and levels of TnT, CK-MB, and LDH were measured by ELISA. **(M)** Analysis of cell viability (CCK-8 assay) in HL-1 cells pre-treated with the STING agonist G10 and subjected to hypoxia for 48 h. **(N)** LDH levels in HL-1 cell culture supernatants were determined by ELISA. #p<0.05.

To assess whether the cardioprotective effect of Lats2 ablation in AMI is dependent on STING, STING agonist-1 (G10) was administered to the mice before AMI induction. Echocardiography data displayed G10 abrogated the normalizing effects of Lats2 deletion on myocardial contraction parameters, as evidenced by impaired LVEF/LVEF, decreased IVS, and increased LVDs (Figure 7A-7G). Likewise, indicators of cardiac muscle relaxation capacity (LVDd, E/A and E/e') were also impaired in *Lats2^Cko^* mice after G10 administration (Figure 7A-7G). G10 treatment negated also the preventative actions of *Lats2* ablation on the transcription of the pro-fibrosis genes MMP9 and TGFβ (Figure 7H, 7I), and reversed AMI-induced upregulation of the cardiomyocyte rupture biomarkers TnT, CK-MB, and LDH in mouse sera (Figure 7J-7L).

Meanwhile CCK-8 and LDH release assays in HL-1 cells *in vitro* showed that the pro-survival effect of *Lats2*/siRNA transfection was abrogated in cells pre-treated with G10 (Figure 7M, 7N). Taken together, these results confirmed our hypothesis that Lats2 deficiency confers cardioprotection by inhibiting STING activation.

## Discussion

The present study explored the molecular basis of Lats2 in AMI. Three main findings could be obtained. First, Lats2 is significantly upregulated in heart tissue after AMI, and its expression correlates with worsened heart function. Second, Lats2 upregulation promoted cardiomyocyte death through accelerating mitochondrial dysfunction and launching the mitochondria-dependent apoptosis signalings. Third, the molecular mechanism underlying Lats2-induced mitochondrial dysfunction and apoptosis is associated with activation of mitochondrial fission and subsequent induction of cGAS/STING/p65 signaling. Based on the above findings, Lats2 upregulation seems to be a critical pathological factor exacerbating myocardial damage during AMI. Therefore, blocking Lats2 upregulation or inactivating the mitochondrial fission/cGAS/STING/p65 pathway may be useful therapeutic strategies to attenuate cardiac damage in AMI.

Mitochondrial abnormalities significantly promote the pathogenesis of AMI, as it leads to reduced ATP metabolism, redox imbalance, and abnormal calcium handling in cardiomyocytes, resulting in myocardial tissue injury and death 45. Our experiments showed that Lats2 ablation *in vivo* ameliorated AMI-related cardiac dysfunction and damage by attenuating mitochondrial dysfunction. Specifically, Lats2 deficiency inhibited mitochondrial fission, prevented mPTP opening and mitochondrial membrane rupture, inhibited the leakage of mtDNA into the cytosol, and blocked apoptosis by inactivating cGAS/STING/p65 signaling. These findings are consistent with previous studies 41, 42. In hepatocellular carcinoma (HCC) cells, Drp1-dependent division aguments mtDNA stress and thus enhances the secretion of CCL2 via the TLR9-involved NF-κB pathway 15. Under physiological conditions, mitochondrial fission/fusion events regulate the localization of mtDNA in the mitochondrial population to sustain an adequate respiratory capacity 46. However, the exact mechanism by which mitochondrial fission promotes mtDNA release into the cytosol is still under investigation. It is thought that fragmentation of mitochondria during fission induce mitochondrial membrane rupture, leading to mtDNA leakage 13-15. Additionally, enhanced mitochondrial fission may facilitate the recruitment of the autophagy machinery to damaged mitochondria, promoting the engulfment and subsequent degradation of the organelles and the release of mtDNA 47, 48. In our studies, we found that in the AMI setting, loss of *Lats2* inhibits mitochondrial fission and thus prevents mtDNA release outside of mitochondria.

The cGAS/STING cascade is characteristically induced in response to the presence of foreign (e.g. viral or bacterial) DNA 49, 50 in the cytoplasm. Notably, this signaling is also sensed by mtDNA upon mitochondrial damage 51, 52. The molecular basis through which mtDNA induces the cGAS/STING cascade involves several steps. First, mtDNA is captured by cGAS, which binds to mtDNA and thus induces cGAMP 53. Next, the crosslink between cGAMP and STING 54 leads to STING activation, which augments the abundance of interferon regulatory factor 3 (IRF3) and NF-κB 55. Nuclear expression of IRF3 and NF-κB leads to inflammation and tissue damage by inducing the transcription of proinflammatory cytokines and interferons. These are typically involved in the inflammation reaction against pathogens 56 and are expressed in the presence of cytosolic mtDNA 57, 58. The present data indicated that mtDNA release, resulting from enhanced mitochondrial fission, determines the opening of the cGAS/STING/p65 cascade in hypoxic cardiomyocytes. Furthermore, evidence that this signaling axis is a downstream effector of Lats2 activation in AMI was obtained *in vivo*, as *Lats2* ablation fully prevented STING activation and relieved heart damage in our AMI model. Accordingly, reactivation of STING signaling through administration of the STING agonist G10 abolished the cardioprotective effects of *Lats2* deletion in mice.

The pathological contribution of the cGAS/STING cascade to cardiovascular disorders is widely described. Angiotensin II-mediated cardiac hypertrophy is associated with STING activation and subsequent inflammation and fibrosis due to ER stress 59. In septic cardiomyopathy, inhibition of the cGAS/STING cascade sustains heart function through preventing NLRP3-mediated apoptosis and pyroptosis 60. Given the necessary actions offered by STING in myocardial inflammation and cardiac apoptosis, the usage of selective or specific STING inhibitors arises as a promising strategy for the treatment of cardiac fibrosis after AMI 61.

Three limitations require further exploration. First, the mechanism by which Lats2 activates STING was not specifically addressed; thus, the potential interaction between Lats2 and STING should be verified. Since Lats2 is a kinase, it is reasonable to guess that cGAS/STING could be a phosphorylation substrate of Lats2. Although the phosphorylation of cGAS/STING is observed by several studies, the identity of the upstream kinase remains unclear 62.

In conclusion, our studies described the pathological role of Lats2 in myocardial dysfunction after AMI. We thus propose that increased expression of Lats2 is a novel phenotypic alteration in the infarcted heart that leads to impaired cardiomyocyte viability and function by inducing mitochondrial dysfunction. Mechanistically, Lats2 promotes mitochondrial fission and thus activates the cGAS/STING/p65 cascade, ultimately contributing to cardiomyocyte death and decreased heart performance after AMI.

## Supplementary Material

Supplementary information.
