# Supplementary material for: Lats2 deficiency protects the heart against myocardial infarction by reducing inflammation and inhibiting mitochondrial fission and STING/p65 signaling
Source: Int J Biol Sci. 2023 Jul 3;19(11):3428–40. doi: 10.7150/ijbs.84426 (PMC10367568; doi:10.7150/ijbs.84426)
Supplement: Supplementary file 1 — Supplementary information. [file ijbsv19p3428s1.pdf]

Supplemental Information

Lats2 deficiency protects the heart against myocardial infarction by reducing inflammation and inhibiting mitochondrial fission and STING/p65 signaling

The following primers were used:
TFAM (Forward, 5'-GGCGAATTCCTCGAGGCCACCATGGCGCTGTTCCGGGGAATGT-3'; Reverse, 5'-CATACGCGTATGCTCAGAGATGTCTCCGGATCGT-3’),
TRMT10C (Forward, 5'-GGAAGCCGTGCTGTAGGA-3'; Reverse, 5'-AGCTGCTCAGGAGGGGAT-3’),
ELAC2 (Forward, 5'-GAGAAGGCGTCCAACGACTTA -3'; Reverse, 5'-AGAAAGATGTTGTCCAAGCGAG-3’),
FASTKD2 (Forward, 5'-AGCTGGTACCATGAATAACAAAGCG-3'; Reverse, 5'-ACGTCTCGAGTTGTGTGCTTTGCAC-3’),
Fis1 (Forward, 5'-GGCTGTCTCCAAGTCCAAATC-3'; Reverse, 5'-GGAGAAAAGGGAAGGCGATG-3’),
Drp1 (Forward, 5'- GGGCACTTAAATTGGGCTCC-3’; Reverse, 5’-TGTATTCTGTTGGCGTGGAAC-3’),
Mff (Forward, 5’-GGCTGTCTCCAAGTCCAAATC-3’; Reverse, 5’-GGAGAAAAGGGAAGGCGATG-3’);
TGFβ (Forward, 5′-TACCATGCCAACTTCTGTCTGGGA-3′; Reverse, 5′-ATGTTGGACAACTGCTCCACCTTG-3′);
MMP9 (Forward, 5′-GGCGAATTCCTCGAGGCCACCATGGCGCTGTTCCGGGGAATGT-3′; Reverse, 5′- CATACGCGTATGCTCAGAGATGTCTCCGGATCGT -3′).
